# Comparison of Unplanned Intensive Care Unit Readmission Scores: A Prospective Cohort Study

**DOI:** 10.1371/journal.pone.0143127

**Published:** 2015-11-23

**Authors:** Regis Goulart Rosa, Cintia Roehrig, Roselaine Pinheiro de Oliveira, Juçara Gasparetto Maccari, Ana Carolina Peçanha Antônio, Priscylla de Souza Castro, Felippe Leopoldo Dexheimer Neto, Patrícia de Campos Balzano, Cassiano Teixeira

**Affiliations:** 1 Department of Critical Care, Hospital Moinhos de Vento, Porto Alegre, Brazil; 2 School of Medicine, Universidade Federal de Ciências da Saúde de Porto Alegre (UFCSPA), Porto Alegre, Brazil; D'or Institute of Research and Education, BRAZIL

## Abstract

**Purpose:**

Early discharge from the intensive care unit (ICU) may constitute a strategy of resource consumption optimization; however, unplanned readmission of hospitalized patients to an ICU is associated with a worse outcome. We aimed to compare the effectiveness of the Stability and Workload Index for Transfer score (SWIFT), Sequential Organ Failure Assessment score (SOFA) and simplified Therapeutic Intervention Scoring System (TISS-28) in predicting unplanned ICU readmission or unexpected death in the first 48 hours after discharge from the ICU.

**Methods:**

We conducted a prospective cohort study in a single tertiary hospital in southern Brazil. All adult patients admitted to the ICU for more than 24 hours from January 2008 to December 2009 were evaluated. SWIFT, SOFA and TISS-28 scores were calculated on the day of discharge from the ICU. A stepwise logistic regression was conducted to evaluate the effectiveness of these scores in predicting unplanned ICU readmission or unexpected death in the first 48 hours after discharge from the ICU. Moreover, we conducted a direct accuracy comparison among SWIFT, SOFA and TISS-28 scores.

**Results:**

A total of 1,277 patients were discharged from the ICU during the study period. The rate of unplanned ICU readmission or unexpected death in the first 48 hours after discharge from the ICU was 15% (192 patients). In the multivariate analysis, age (*P* = 0.001), length of ICU stay (*P* = 0.01), cirrhosis (*P* = 0.03), SWIFT (*P* = 0.001), SOFA (*P* = 0.01) and TISS-28 (*P*<0.001) constituted predictors of unplanned ICU readmission or unexpected death. The SWIFT, SOFA and TISS-28 scores showed similar predictive accuracy (AUC values were 0.66, 0.65 and 0.74, respectively; *P* = 0.58).

**Conclusions:**

SWIFT, SOFA and TISS-28 on the day of discharge from the ICU have only moderate accuracy in predicting ICU readmission or death. The present study did not find any differences in accuracy among the three scores.

## Introduction

Rates of intensive care unit (ICU) readmission have become a metric of hospital and provider performance as well as a means by which to incentivize efficient, high quality, and coordinated patient care [1]. The Quality Indicators Committee of the Society of Critical Care Medicine has stated that readmission within 48 hours is a major performance indicator of the quality of intensive care medicine [2,3]

Prolonged duration of stay in an ICU is costly, stressful for patients and families, reduces the number of beds available for other patients, and can increase risk for iatrogenic and nosocomial complications [4]. However, early discharge from the ICU is not without risk. If patients requiring high intensity care are discharged before they can be safely cared for in a lower-acuity care environment, they are at risk for both complications and delayed recognition of clinical deterioration. The former can result in the need for unplanned ICU readmission; the latter can result in patient death [5,6]. In addition, ICU readmission also places additional stress on patients, ICU staff and families.

Risk stratification of patients discharged from the ICU is a complex process with many potential challenges. Several risk stratification tools have been developed; however, at present it is unclear whether the existing tools provide value above clinical judgment or whether they can be used to improve healthcare delivery [7–14]. Previously identified predictors of death or ICU readmission include duration of ICU stay, Glasgow Coma Scale at the time of ICU discharge, mean arterial blood pressure, and ICU admission source [15]. Others have attempted to create decision support tools to assist in ICU discharge readiness assessment [16]. The Sequential (sepsis-related) Organ Failure Assessment score (SOFA) is used to track a patient’s status during admission to the ICU. SOFA is a scoring system primarily designed to determine the extent of a person’s organ function or rate of failure, not to predict ICU readmission [17,18]. The Stability and Workload Index for Transfer score (SWIFT), which was developed to predict readmission or death within 1 week of ICU discharge, has demonstrated only moderate discrimination power to predict these [14].

In addition to the severity of illness score, there is also an association between nursing workload and post-ICU mortality [19,20]. The simplified Therapeutic Intervention Scoring System (TISS-28) has been widely applied to assess workload and resource allocation in intensive care, thereby measuring treatment intensity [4]. Several authors [19–21] have shown an association between the TISS-28 value on the last ICU day and post-ICU mortality, and therefore, an indirect association with ICU readmission.

Unfortunately, there are few data available regarding a comparison among distinct ICU readmission scores. Accordingly, we performed the present study to compare the effectiveness of SWIFT, SOFA and TISS-28 scores in predicting early unplanned ICU readmission or death after discharge from the ICU.

## Methods

### Study design, patients and setting

A prospective cohort study was conducted at a single tertiary centre. The present study followed all intensive care patients >18 years of age who were consecutively discharged from the 31-bed mixed medical-surgical ICU of the Hospital Moinhos de Vento in Porto Alegre, Brazil, from January 2008 to December 2009. Subjects who had an ICU length of stay <24 hours were excluded. Patients were not allowed to reenter the study after a first episode of ICU admission.

### Definitions

The main independent variables were SWIFT, SOFA and TISS-28 scores calculated on the day of discharge from the ICU by researchers who were not associated with the attending physician’s team. Elements of the SWIFT score include ICU admission source, ICU length of stay, day of discharge GCS, last PaO_2_/FiO_2_ ratio, and last arterial blood gas PaCO_2_ (Table 1) [14]. The SOFA score is based on extent of the patient’s organic function determined by physiological parameters of respiratory, cardiovascular, hepatic, coagulation, neurologic, and renal systems (Table 2) [17]. The TISS-28 score comprises interventions related to basic activities, cardiovascular support, specific interventions, ventilator support, renal support, neurologic support, and metabolic support (Table 3) [22].

**Table 1:** SWIFT (stability and workload index for transfer).

Variable	SWIFT Points
**Original source of ICU admission**	
Emergency department.	0
Transfer from a ward or outside hospital.	8
**Total ICU length of stay**	
< 2 days.	0
2 to 10 days.	1
> 10 days.	14
**Last measured PaO**_**2**_ **/FiO**_**2**_ **ratio**	
≥ 400.	0
< 400.	5
< 150.	10
< 100.	13
**Glasgow coma scale at time of ICU discharge**	
≥ 14.	0
11 to 14.	6
8 to 11.	14
< 8.	24
**Last arterial blood gas PaCO**_**2**_	
≤ 45 mmHg	0
> 45 mmHg	5

Notes: Data taken from Gajic et al. [14]. SWIFT score = sum of SWIFT points.

**Table 2:** SOFA (sequential organ failure assessment).

Variable	SOFA Points
**Respiratory**	
PaO_2_/FiO_2_ ratio < 400 +/- respiratory support.	1
PaO_2_/FiO_2_ ratio < 300 +/- respiratory support.	2
PaO_2_/FiO_2_ ratio < 200 and respiratory support.	3
PaO_2_/FiO_2_ ratio < 200 and respiratory support.	4
**Cardiovascular**	
MAP < 70 mmHg.	1
Dopamine ≤ 5 mcg/Kg/min or dobutamine (any dose).	2
Dopamine > 5 mcg/Kg/min or epinephrine ≤ 1 mcg/Kg/min or norepinephrine ≤ 1 mcg/Kg/min.	3
Dopamine > 15 mcg/Kg/min or epinephrine > 1 mcg/Kg/min or norepinephrine > 1 mcg/Kg/min.	4
**Liver**	
Serum bilirubin 1.2 to 1.9 mg/dL.	1
Serum bilirubin 2.0 to 5.9 mg/dL.	2
Serum bilirubin 6.0 to 11.9 mg/dL	3
Serum bilirubin > 12.0 mg/dL.	4
**Coagulation**	
Platelets count < 150,000 /mm^3^.	1
Platelets count < 100,000 /mm^3^.	2
Platelets count < 50,000 /mm^3^.	3
Platelets count < 20,000 /mm^3^.	4
**Glasgow Coma Score**	
13 to 14.	1
10 to 12.	2
6 to 9.	3
< 6.	4
**Renal**	
Serum creatinine 1.2 to 1.9 mg/dL.	1
Serum creatinine 2.0 to 3.4 mg/dL.	2
Serum creatinine 3.5 to 4.9 mg/dL or urine output < 500 mL/day.	3
Serum creatinine > 5.0 mg/dL or urine output < 200 mL/day.	4

Notes: Data taken from Vincent et al. [17]. SOFA score = sum of SOFA points.

**Table 3:** TISS-28 (therapeutic intervention scoring system).

Category	TISS-28 Points
**Basic Activities**	
Standard monitoring (hourly vital signs, fluid balance).	5
Biochemical and microbiological investigations.	1
Singe medication (any route).	2
Multiple intravenous medications.	3
Care and prevention of decubitus and daily dressing changes.	1
Frequent dressing changes (at least one time per each nursing shift).	1
Care of drains.	3
**Cardiovascular Support**	
Single vasoactive medication.	3
Multiple vasoactive medications.	4
Intravenous replacement of large fluid losses (> 3 L/m^2^/day).	4
Peripheral arterial catheter.	5
Pulmonary artery flotation catheter.	8
Central venous line.	2
Cardiopulmonary resuscitation after arrest in the past 24 hours.	3
**Specific interventions**	
Single specific interventions in the ICU (naso or orotracheal intubation, cardioversion, introduction of peacemaker, endoscopies, emergency surgery in the past 24 hours).	3
Multiple specific interventions in the ICU (more than one described above).	5
Specific interventions outside ICU (surgery or diagnostic procedures).	5
**Ventilatory Support**	
Mechanical ventilation.	5
Supplementary ventilation support (supplementary oxygen by any method except if mechanical ventilation parameters apply).	2
Care of artificial airways (endotracheal tube or tracheostoma).	1
Treatment for improving lung function (e.g. thorax physiotherapy, incentive spirometry, inhalation therapy, intratracheal suctioning).	1
**Renal Support**	
Hemofiltration/dialytic techniques.	3
Quantitative urine output measurement.	2
Active diuresis (e.g. furosemide > 0.5 mg/Kg/day).	3
**Neurologic Support**	
Measurement of intracranial pressure.	4
**Metabolic Support**	
Treatment of complicated metabolic acidosis/alkalosis.	4
Intravenous alimentation.	3
Enteral feeding through gastric tube or other route (e.g. jejunostomy).	2

Notes: Data taken from Moreno et al. [22]. TISS-28 score = sum of TISS-28 points.

Other covariates analysed included age, sex, type of comorbidity, need for and length of mechanical ventilation, need for tracheostomy, and length of ICU stay.

### Outcomes and follow-up

The primary outcomes of the study were unplanned ICU readmission or unexpected death in the first 48 hours after discharge from the ICU. The 48 hours cut-off for readmission or death was chosen because this timeframe is often accepted for evaluating the quality of ICU discharge; given that earlier the readmission or death, the more likely the patient was unprepared to be discharge from ICU. [2,3].

Patients were followed up through interviews and medical record reviews using a standardised case report form, by researchers who were not associated with the attending physician’s team. Follow-up was maintained for 48 hours after discharge from the ICU.

### Statistical analysis

A stepwise backward multivariate logistic regression was performed to determine whether SWIFT, SOFA and TISS-28 scores were predictors of unplanned ICU readmission or unexpected death in the first 48 hours after discharge from the ICU. All variables with *P*<0.15 in the univariate analysis were included. In the multivariate model, independent variables were eliminated from the highest to the lowest *P*-value, but retained in the model if *P<*0.05. Odds ratios (ORs) were estimated along with the 95% confidence intervals (CIs). The accuracy of different score systems for predicting unplanned ICU readmission or unexpected death in the first 48 hours after ICU discharge was evaluated through the area under the receiver operating characteristic (ROC) curve (AUC). Values of the AUC greater than 0.8 were considered good, between 0.6–0.8 moderate, and lower than 0.6 poor for prediction performance. Univariate AUCs modeling of the three scores were compared using the chi-squared test. Observed and predicted unplanned ICU readmission or unexpected death in the first 48 hours after discharge from the ICU were compared using the Hosmer-Lemeshow test. Stata Statistical Software Release 12 (StataCorp LP, College Station, TX, USA) was used for statistical analysis.

### Ethics issues

The Institutional Review Board of the Hospital Moinhos de Vento approved the study, and written informed consent was obtained from all study participants.

## Results

During the study period, 1,277 patients were discharged from the ICU. The characteristics of study population are summarized in Table 4. The mean age of the cohort was 67 years. The mean Apache-II and SOFA scores on the day of ICU admission were 15.4 and 2.8, respectively. The predominant underlying comorbidities were ischemic heart disease (25.5%), diabete mellitus (22.0%), malignant neoplasia (21.0)%, peripheral vascular disease (13.0%), chronic obstructive pulmonary disease (11.9%) and heart failure (11.5%). Surgical patients comprised 39.3% of the study population. Mechanical ventilation was needed in 27.7% of patients and the mean length of mechanical ventilation was 5.3 days. The mean length of ICU stay was 7.3 days. On the day of discharge from ICU the mean SWIFT, SOFA and TISS-28 scores were 12.0, 1.0 and 11.5, respectively. All patients were directly discharged to general medical or surgical wards without hospitalization in intensive care step-down units.

**Table 4:** Characteristics of 1,277 adult patients discharged from ICU.

Age, mean years, mean (SD)	67.2 (17.7)
Male sex	688 (53.8)
APACHE-II score 24 hours after ICU admission, mean (SD)	15.4 (6.8)
SOFA score at ICU admission, mean (SD)	2.8 (3.0)
TISS-28 score 24 hours after ICU admission, mean (SD)	18.8 (8.1)
Comorbitities	
Ischemic heart disease	326 (25.5)
Heart failure	147 (11.5)
Diabete mellitus	281 (22.0)
Chronic obstructive pulmonary disease	152 (11.9)
Hemodialysis	77 (6.0)
Malignant neoplasia	269 (21.0)
Peripheral vascular disease	167 (13.0)
Cirrhosis	29 (2.2)
Neuromuscular disease	15 (1.1)
Surgical patients	
Elective	411 (32.1)
Urgent	93 (7.2)
Mechanical ventilation during ICU stay	354 (27.7)
Duration of mechanical ventilation, days, mean (SD)	5.3 (9.1)
Tracheostomy	42 (3.2)
Length of ICU stay, days, mean (SD)	7.3 (12.1)
Day of discharge SWIFT score, mean (SD)	12.0 (8.3)
Day of discharge SOFA score, mean (SD)	1.01 (1.6)
Day of discharge TISS-28 score, mean (SD)	11.5 (3.2)

Note: Data presented as n (%) unless otherwise indicated. Abbreviations: SD, standard deviation; APACHE-II, acute physiology and chronic Health Evaluation; SOFA, sequential organ failure assessment score; TISS-28, simplified therapeutic intervention scoring system; SWIFT, stability and workload index for transfer score.

The overall rate of unplanned ICU readmission or unexpected death in the first 48 hours after ICU discharge was 15.0% (192 patients). Of these 126 patients (65.6%) had unplanned ICU readmission and 66 patients died unexpectedly (34.4%). The main reasons for ICU readmission were acute respiratory failure (46.0%), sepsis (30.9%), cardiac conditions such as arrhythmia and congestive heart failure (15.8%) and neurologic impairment (7.1%). Among those patients who died unexpectedly, 31% had the “do not resuscitate” order at ICU discharge.

In the univariate analysis of risk factors for unplanned ICU readmission or unexpected death in the first 48 hours after ICU discharge (Table 5), age (*P*<0.001), previous heart failure (*P* = 0.004), previous chronic obstructive pulmonary disease (*P* = 0.02), previous cancer (*P* = 0.01), previous cirrhosis (*P* = 0.01), renal replacement therapy (*P* = 0.01), mechanical ventilation required during ICU stay (*P*<0.001), duration of mechanical ventilation (*P*<0.001), need for tracheostomy (*P* = 0.001), length of ICU stay (*P*<0.001), SWIFT (*P*<0.001), SOFA (*P*<0.001) and TISS-28 (*P*<0.001) scores were positively associated with the main outcomes. After multivariate analysis was conducted (Table 6), variables that constituted independent risk factors for unplanned ICU readmission or unexpected death in the first 48 hours after discharge from the ICU included age (OR, 1.01; 95% CI, 1.006–1.028), length of ICU stay (OR, 1.01; 95% CI, 1.003–1.030), previous cirrhosis diagnosis (OR, 2.70; 95% CI, 1.06–6.84), SWIFT (OR, 1.03; 95% CI, 1.01–1.06), SOFA (OR, 1.12; 95% CI, 1.02–1.24) and TISS-28 (OR, 1.12; 95% CI, 1.06–1.18) scores.

**Table 5:** Univariate logistic regression of factors associated with unplanned intensive care unit (ICU) readmission or death.

Variable	No readmission to ICU (n = 1085)	Unplanned ICU readmission or death (n = 192)	OR (95% CI)	*P*-value
Age, years, mean (SD)	66.3 (17.6)	72.1 (17.9)	1.02 (1.01–1.03)	<0.001
Male sex	576 (53.0)	112 (58.3)	1.23 (0.90–1.68)	0.17
Ischemic heart disease	281 (25.8)	45 (23.4)	0.87 (0.61–1.25)	0.47
Heart failure	113 (10.4)	34 (17.7)	1.85 (1.21–2.81)	0.004
Diabete mellitus	237 (21.8)	44 (22.9)	1.06 (0.73–1.53)	0.74
Chronic pulmonar obstructive disease	120 (11.0)	32 (16.6)	1.60 (1.05–2.45)	0.02
Hemodialysis	58 (5.3)	19 (9.8)	1.94 (1.13–3.34)	0.01
Malignant neoplasia	216 (19.9)	53 (27.6)	1.53 (1.08–2.17)	0.01
Peripheral vascular disease	139 (12.8)	28 (14.5)	1.16 (0.74–1.80)	0.50
Cirrhosis	20 (1.8)	9 (4.6)	2.61 (1.17–5.84)	0.01
Neuromuscular disease	12 (1.1)	3 (1.5)	1.41 (0.39–5.07)	0.59
Mechanical ventilation during ICU stay	276 (25.4)	78 (40.6)	2.0 (1.45–2.75)	<0.001
Duration of mechanical ventilation, days, mean (SD)	4.6 (8.0)	7.6 (11.8)	1.04 (1.02–1.07)	<0.001
Tracheostomy	28 (2.5)	14 (7.3)	2.96 (1.53–5.74)	0.001
Length of ICU stay, days, mean (SD)	6.2 (9.4)	13.5 (20.8)	1.03 (1.02–1.05)	<0.001
Day of discharge SWIFT score, mean (SD)	11.2 (7.6)	16.8 (10.4)	1.07 (1.05–1.09)	<0.001
Day of discharge SOFA score, mean (SD)	0.9 (1.5)	1.6 (1.7)	1.24 (1.14–1.34)	<0.001
Day of discharge TISS-28 score, mean (SD)	11.2 (3.0)	13.4 (4.0)	1.20 (1.15–1.26)	<0.001

Note: Data presented as n (%) unless otherwise indicated. Abbreviations: OR, odds ratio; 95% CI, 95% confidence interval; SD, standard deviation; SWIFT, stability and workload index for transfer score; SOFA, sequential organ failure assessment score; TISS-28, simplified therapeutic intervention scoring system.

**Table 6:** Multivariate logistic regression model of factors associated with unplanned intensive care unit (ICU) readmission or death.

Variable	Adjusted OR	95% CI	*P*-value
Age, per year	1.01	1.006–1.028	0.001
Length of ICU stay, per day	1.01	1.003–1.030	0.01
Cirrhosis	2.70	1.069–6.840	0.03
Day of discharge SOFA score, per point	1.12	1.025–1.243	0.01
Day of discharge TISS-28 score, per point	1.12	1.064–1.184	<0.001
Day of discharge SWIFT score, per point	1.03	1.015–1.061	0.001

Abbreviations: OR, odds ratio; 95% CI, 95% confidence interval; SWIFT, stability and workload index for transfer score; SOFA, sequential organ failure assessment score; TISS-28, simplified therapeutic intervention scoring system.

**Fig 1:**
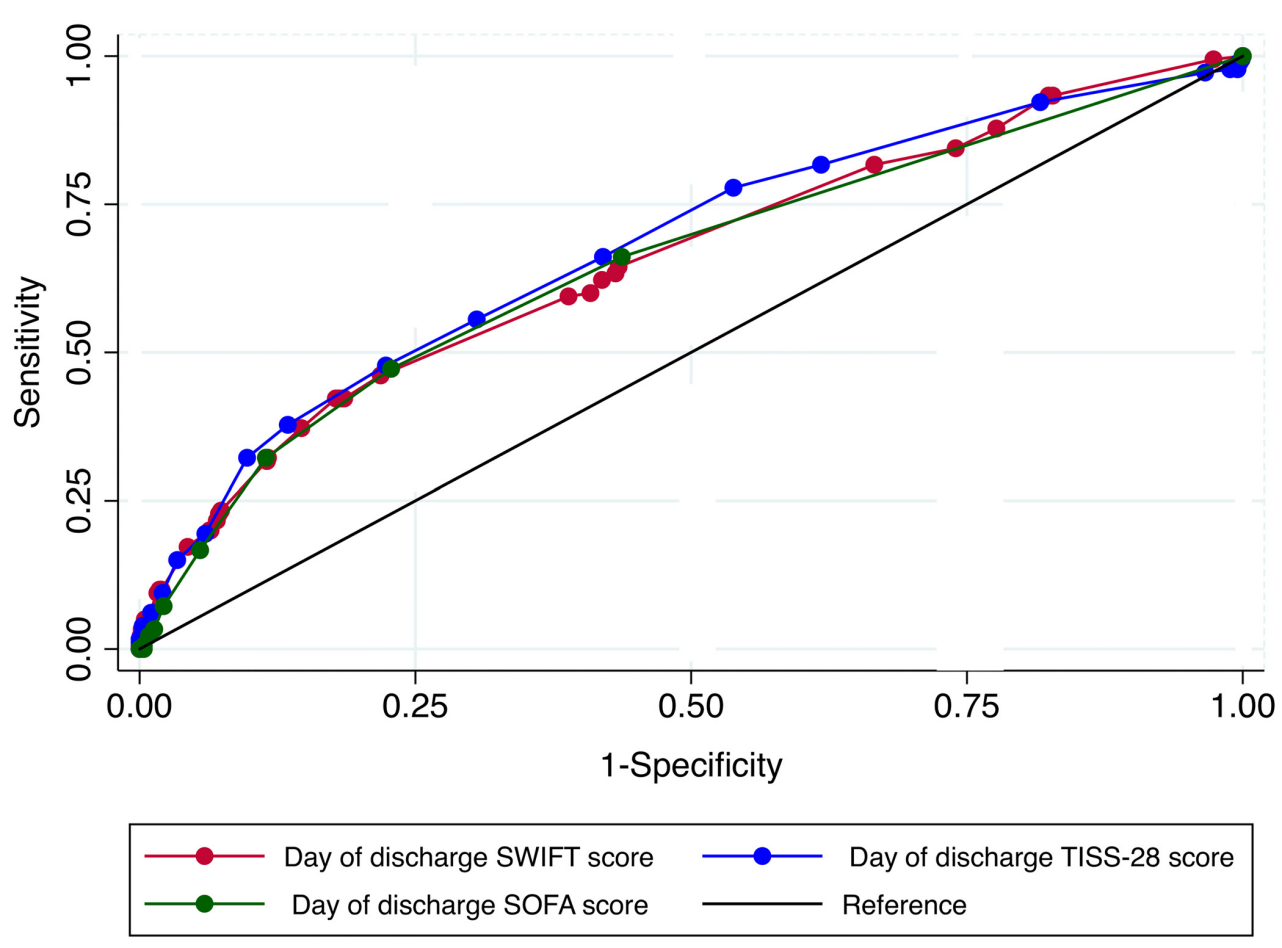
Comparison of receiver operating characteristic (ROC) curves for unplanned intensive care unit readmission or death among SWIFT, SOFA and TISS-28 scores.

The accuracy analysis of SWIFT, SOFA and TISS-28 scores showed only moderate discrimination power to predict unplanned ICU readmission or unexpected death in the first 48 hours after discharge from the ICU, for all three scores (Table 7, Fig 1). The Hosmer-Lemeshow *P*-values for SWIFT, SOFA and TISS-28 scores were 0.66, 0.65 and 0.74, respectively, showing good calibration of the three predictive scores. A direct accuracy comparison among the three scores showed no statistical difference. Table 8 shows the relationship between sensitivity and specificity according to determined cutoffs for SWIFT, SOFA and TISS-28 scores.

**Table 7:** Comparison of predictive accuracy for unplanned intensive care unit readmission or death among SWIFT, SOFA and TISS-28 scores.

Score	Number of observations	AUC	SE	95% CI
Day of discharge SWIFT score	1237	0.65	0.02	0.61–0.70
Day of discharge SOFA score	1277	0.65	0.02	0.60–0.69
Day of discharge TISS-28 score	1277	0.67	0.02	0.63–0.72

Notes: H0: SWIFT = SOFA = TISS-28; Chi-squared test = 1.06; *P*-value = 0.58. Abbreviations: AUC, area under receiver operating characteristic curve; SE, standard error; 95%CI, 95% confidence interval; SWIFT, stability and workload index for transfer score; SOFA, sequential organ failure assessment score; TISS-28, simplified therapeutic intervention scoring system.

**Table 8:** Sensitivity and specificity for unplanned intensive care readmission or death according to determined cutoffs for SWIFT, SOFA and TISS-28 scores.

	Sensitivity	Specificity
**Day of discharge SWIFT score**		
≥ 7 points	88.0%	22.2%
≥ 16 points	42.7%	81.1%
**Day of discharge SOFA score**		
≥ 1 points	62.3%	56.9%
≥ 3 points	30.3%	88.6%
**Day of discharge TISS-28 score**		
≥ 10 points	81.6%	38.2%
≥ 16 points	32.2%	90.2%

Abbreviations: SWIFT, stability and workload index for transfer; SOFA, sequential organ failure assessment; TISS-28, simplified therapeutic intervention scoring system.

## Discussion

In the present study, SWIFT, SOFA and TISS-28 scores evaluated on the day of discharge from the ICU were independently associated with unplanned ICU readmission or unexpected death in the first 48 hours after ICU discharge; however, all three scores showed only moderate predictive accuracy. Additionally, this study failed to find any accuracy difference among SWIFT, SOFA and TISS-28 scores in the ability to predict unplanned ICU readmission or unexpected death in the first 48 hours after discharge from the ICU.

Previous publications have shown promising results of using the SWIFT score to predict unplanned ICU readmission [14,23,24]. For example, Gagic et al. [14] demonstrated the superiority of the SWIFT score, compared with APACHE III, in predicting unplanned ICU readmission (AUC 0.75 [95% CI, 0.70–0.80] versus AUC 0.62 [95% CI, 0.56–0.68]). Moreover, Oakes et al. [23] found good predictive accuracy for SWIFT (AUC 0.76 [95% CI, 0.61–0.91]) in a small sample of ICU patients in southern Brazil. However, in our cohort, the SWIFT, a tool designed specifically to predict unplanned ICU readmission, was not superior to conventional scores of clinical severity (SOFA) or loading interventions (TISS-28) on the day of discharge from the ICU. Congruent to our results, the retrospective study of Kastrup et al. [24] concluded that the SWIFT score was not advantageous when deciding whether a patient can be safely discharged from the ICU, owing to its poor accuracy (AUC 0.58 [95% CI, 0.55–0.60]).

Our findings reinforce that, at present, it is unclear whether existing ICU readmission scores provide value above clinical judgment or whether they can be used to improve outcomes in patient care transition scenarios. Similarly, a systematic review of tools for predicting severe adverse events following patient discharge from the ICU concluded that further evaluation of existing ICU readmission scores is required prior to clinical implementation, given that it is unclear whether a reliable and valid risk stratification tool for patient ICU discharge has been developed [25]. We hypothesise that unplanned ICU readmission or unexpected death is not fully explained by patient characteristics such as extent of organ dysfunction. Organizational aspects of care following ICU discharge should be incorporated into the predictive equation of unplanned readmission. For example, providing step-down units (e.g., respiratory or intermediate care units), for ICU-discharged patients who need more intensive monitoring and rehabilitation care than can be provided in a general medical ward, might be a good way to avoid unexpected clinical deterioration in some patients following ICU discharge [26]. Perhaps we are focusing too much on patient characteristics and ICU needs, and forgetting the type of hospital care required after discharge from the ICU [27,28].

It is interesting to note that the three tools (SWIFT, SOFA and TISS-28), which were developed for different purposes, demonstrated the same accuracy in assessing the outcomes studied. SOFA was designed to determine the extent of a person’s organ function or rate of failure, SWIFT was developed to predict readmission or death within 1 week of ICU discharge, and TISS-28 is applied to assess workload and resource allocation in intensive care, measuring treatment intensity [4,14,17,18]. In our opinion, these findings represent the current lack of understanding regarding the pathophysiological mechanisms of clinical deterioration in patients and need for correct identification of risk factors that accurately reflect the need for readmission of patients to an ICU.

The present study had some limitations. Our rates of ICU readmission in the first 48 hours after discharge from the ICU were higher than in previous reports from ICUs in Europe and North America, which have ICU readmission rates around 2–5% [6,9,10,14,15]. For example, the study of Badawi et al. found rates of readmission and death within 48hs of ICU discharge of 2.5% and 0.9%, respectively, in a robust cohort of more than 700,000 ICU patients [29]. These differences are possibly owing to intensive care practice in Brazil, which is characterized by difficulties in establishing exclusive palliative care during and after ICU discharge and a lack of step-down units for selected patients discharged from the ICU. In addition, assessment of patients only in the first 48 hours after discharge from the ICU may cause difficulties in generalizing the findings of the study, given that previous data suggest that fewer than approximately 50% of ICU readmissions occur less than 48 hours after discharge [6,9]. Another 25% of readmissions occur between 2 and 7 days after ICU discharge; these data were not evaluated in our study. Afternoon and evening discharge are important risk factors for ICU-readmission as well and were not evaluated here. Furthermore, approximately 60% of patients are readmitted for different diagnoses than their original diagnosis, an impossible risk to measure [6,9,16]. Nevertheless, the possibility of systematic errors was minimized by proper measurement of variables and outcomes using previously defined objective criteria, the use of standardized data collection, and prospective follow-up performed by a research team that was not involved in patient care.

Future research should explore how patients’ severity of illness at ICU discharge (rather than at ICU admission), as well as floor-based rather than ICU-based organizational structures, contribute to ICU readmission risk. Future research should also examine whether decision making by residents influences ICU readmission rates.

## Conclusions

We conclude that SWIFT, SOFA and TISS-28 scores evaluated on the day of discharge from the ICU can be used to predict unplanned ICU readmission or unexpected death in the first 48 hours after discharge from the ICU, however, with only moderate predictive accuracy.

## Supporting Information

S1 Dataset (XLSX)
