# Correction: Comparison of Unplanned Intensive Care Unit Readmission Scores: A Prospective Cohort Study

**DOI:** 10.1371/journal.pone.0148834

**Published:** 2016-02-05

**Authors:** Regis Goulart Rosa, Cintia Roehrig, Roselaine Pinheiro de Oliveira, Juçara Gasparetto Maccari, Ana Carolina Peçanha Antônio, Priscylla de Souza Castro, Felippe Leopoldo Dexheimer Neto, Patrícia de Campos Balzano, Cassiano Teixeira

There is an error in the last sentence of the Results subsection of the Abstract. The correct sentence is: The SWIFT, SOFA and TISS-28 scores showed similar predictive accuracy (AUC values were 0.66, 0.65 and 0.67, respectively; *P* = 0.58)
